# Differential Modulation of ATP-Induced P2X7-Associated Permeabilities to Cations and Anions of Macrophages by Infection with *Leishmania amazonensis*

**DOI:** 10.1371/journal.pone.0025356

**Published:** 2011-09-23

**Authors:** Camila Marques-da-Silva, Mariana Martins Chaves, Juliany Cola Rodrigues, Suzana Corte-Real, Robson Coutinho-Silva, Pedro Muanis Persechini

**Affiliations:** 1 Instituto de Biofisica Carlos Chagas Filho, Universidade Federal do Rio de Janeiro, Rio de Janeiro, Rio de Janeiro, Brazil; 2 Instituto Oswaldo Cruz, Fundação Oswaldo Cruz, Rio de Janeiro, Rio de Janeiro, Brazil; University Paris Sud, France

## Abstract

*Leishmania* and other parasites display several mechanisms to subvert host immune cell function in order to achieve successful infection. The ATP receptor P2X7, an agonist-gated cation channel widely expressed in macrophages and other cells of the immune system, is also coupled to inflammasome activation, IL-1 beta secretion, production of reactive oxygen species, cell death and the induction of the permeabilization of the plasma membrane to molecules of up to 900 Da. P2X7 receptors can function as an effective microbicidal triggering receptor in macrophages infected with several microorganisms including *Mycobacteria tuberculosis*, *Chlamydia* and *Leishmania*. We have previously shown that its expression is up-regulated in macrophages infected with *L.amazonensis* and that infected cells also display an increase in P2X7-induced apoptosis and membrane permeabilization to some anionic fluorescent dyes. In an independent study we recently showed that the phenomenon of macrophage membrane permeabilization can involve at least two distinct pathways for cations and anions respectively. Here, we re-addressed the effects of ATP-induced P2X7-associated phenomena in macrophages infected with *L.amazonensis* and demonstrated that the P2X7-associated dye uptake mechanisms are differentially modulated. While the membrane permeabilization for anionic dyes is up-modulated, as previously described, the uptake of cationic dyes is strongly down-modulated. These results unveil new characteristics of two distinct permeabilization mechanisms associated with P2X7 receptors in macrophages and provide the first evidence indicating that these pathways can be differentially modulated in an immunologically relevant situation. The possible importance of these results to the *L.amazonensis* escape mechanism is discussed.

## Introduction

Macrophages display strong differentiation plasticity characterized by the modulation of the expression of several membrane receptors, cytokine production, and functions such as migration, phagocytosis, microorganism killing and antigen presentation [1]. These cells are also the host of a number of intracellular pathogens such as *Mycobacterium tuberculosis*, *Leishmania*, *Trypanosoma cruzi*, and HIV-1 [2]–[12]. Therefore, it is no surprise that receptor-agonist interactions are important for macrophage-mediated immune regulation involved in both the resolution of the infection and the survival strategies of the pathogens [13]–[15].

The P2X7 receptor is a member of the P2X ligand-gated cation-selective ion channels that are highly expressed in macrophages, dendritic cells and other cells of the immune system [16]–[21]. Besides its ion-channel activity, it also induces the so-called “permeabilization phenomenon” originally characterized by the uptake of fluorescent molecules of up to 900 Da [22]–[24]. In macrophages, P2X7 receptors have been implied in cell death, inflammasome activation, secretion of IL1β, modulation of regulatory T cell function, and in the killing of intracellular microorganisms, among other effects [21], [25]–[30].

The protozoan *Leishmania amazonensis* (*L.amazonensis*) is an obligatory intracellular parasite that survives and proliferates inside parasitophorous vacuoles (PVs) that reside in the cytosol of its main host cell, the macrophage [31]. Inside PVs, the parasites must subvert several macrophage functions to provide a regular supply of nutrients and avoid being killed by the host cells. In order to do so, they modulate the traffic of endocytic-derived and intracellular vesicles and their fusion with the PVs, while also modulating the expression of membrane proteins derived from both the host and the parasites [32].

Our laboratory recently reported that macrophages infected with *L.amazonensis* up-regulate the expression of functional P2X7 receptors and the ATP-induced permeabilization to the vital dye Lucifer Yellow (LY) [33]. Extracellular ATP (ATPe) also induces macrophage apoptosis and parasite elimination *in vitro* [33].

The increase in permeabilization, a P2X7-associated phenomena that can potentially eliminate the parasite is in apparent conflict with the development of successful escape mechanisms that will ultimately guarantee their survival inside the host cell. The resolution of this conflict may lead us to a better understanding of the P2X7-associated phenomena such as the ATP-induced permeabilization in macrophages and their modulation by the parasite. Two mechanisms have been proposed to explain how P2X7 receptors control the uptake of high weight dyes: one involving the passage of large molecules through the P2X7 channels after a structural modification called “pore dilation”, and the other involving the opening of a second, larger and molecularly distinct pore such as the Z pores we have previously described [22], [23], [34]. Recently, the independent pore hypothesis was favored by the demonstration that in macrophages, astrocytes, and some P2X7-transfected cells, pannexin-1 pores can be formed under the control of agonist binding to P2X7 molecules [35], [36]. However, the permeabilization phenomenon of macrophages proved to be more complex than previously thought since we and others have shown that large organic cationic and anionic dyes are taken up by macrophages through at least two different mechanisms [37], [38]. These recent findings prompted us to hypothesize that the P2X7 receptor and the different transport mechanisms associated with its activation could be differentially modulated during the course of many physiologically-relevant immune-pathological or immunomodulatory processes. In this work we investigated the effects of ATPe on the uptake of cationic and anionic dyes by *L.amazonensis*-infected intraperitoneal murine macrophages and describe for the first time a differential modulation of the P2X7-mediated ATPe-induced uptake of cationic and anionic dyes in an immunologically relevant situation.

## Materials and Methods

### Chemicals

Dulbecco's Modified Eagle's Medium (DMEM), 199 medium, fetal bovine serum (FBS), penicillin, and streptomycin were obtained from Gibco/BRL (São Paulo, SP, Brasil). Fura-2-AM, Hoechst 33342, and probenecid were from Molecular Probes (Eugene, OR, USA). Annexin-V was from Trevigen (Gaithersburg, MD, USA). ATP, ethidium bromide (EB), propidium iodine, sulforhodamine B (SR-B), Lucifer Yellow lithium salt (LY), carboxyfluorescein (CF), oxidized ATP (oxATP), Dextran-FITC (mol wt 10.000), Destran-Rhodamine (mol wt 10,000), Triton X-100, Hemin and Bovine Serum Albumin (BSA) were purchased from Sigma-Aldrich (St Louis, MO, USA).

### Ethics Statement

This study was carried out in strict accordance with the recommendations in the Guide for the Care and Use of Laboratory Animals of the National Institutes of Health (USA). The protocol was approved by the Committee on the Ethics of Animal Experiments of the Health Science Center of the Federal University of Rio de Janeiro (CEUA-CCS, Permit Number: IBCCF 031) and all efforts were made to minimize suffering.

### Animals

Balb/c mice were obtained from the animal facilities of the Instituto de Microbiologia Paulo de Goes and from the Transgenic Animal Facility of the Instituto de Biofísica Carlos Chagas Filho of the Federal University of Rio de Janeiro. All animals were 8- to 12-weeks old, weighed approximately 16–30 g. and were handled according to the guidelines for animal use in scientific experiments of the Instituto de Biofísica Carlos Chagas Filho of the Federal University of Rio de Janeiro.

### Parasites and Macrophages

*L.amazonensis* promastigotes (MHOM/BR/Josefa) were grown in 199 medium supplemented with 10% heat-inactivated FBS and 5% hemin at 24°C. The parasites were used in the late stationary phase. The resident macrophages were obtained from BALB/c mouse peritoneum and plated directly into 24-well plastic dishes (Falcon, Becton Dickinson Labware) or in glass coverslips at a cell density of 5×10^5^ cells per well. After 1 hour, cells were washed gently twice with phosphate buffered saline (PBS) to remove non-adherent cells. Adhered cells were cultured in DMEM supplemented with 10% fetal bovine serum and 100 units Penicillin/Streptomycin at 37°C under a 5% CO_2_ atmosphere. Macrophages were infected with late stationary phase *L.amazonensis* promastigotes at a ratio of 10∶1 (parasite∶macrophage). After 4 h, free parasites were washed off macrophage monolayers with PBS.

Fluorescent parasites were obtained by incubating promastigotes with Dextran -FITC or Dextran- Rhodamine (1 µg/mL) for 2 hours at 27°C in M199 media. Parasites were then washed 3 times in PBS at room temperature, and infection and dye uptake assays were performed as describe below.

In some experiments, mice were intraperitoneally infected with 5×10^7^ parasites in 1 mL sterile PBS for 5 days, the cells were collected by peritoneal wash and dye uptake assays were performed by flow cytometry in freshly isolated cells in suspension.

To obtain dead parasites, promastigotes were washed three times in PBS at room temperature and either submitted to four cycles of freezing-and-thawing in liquid nitrogen-37°C or fixed in 5% paraformaldehyde for 30 minutes at room temperature. The parasites were then washed three times with cold PBS and maintained at 4°C until use.

### Measurement of cell death

To evaluate the sensibility of infected or non-infected macrophages to lysis by ATPe, cells were infected or not, as described above, and after 48 hours they were treated with zero or 3 mM ATP in a solution containing in mM: 145 NaCl, 5 KCl, 1 MgCl_2_, and 10 Na-HEPES, pH 7.4 (normal extracellular solution) for 30 minutes at 37°C in 5% CO_2_. Cells were then gently washed in the same solution without ATP, placed in complete cell culture medium for 10 hours at 37°C in 5% CO_2_ and analyzed for apoptosis or necrosis as described [28]. In brief, the culture plates were first spun down for 10 min at 200× g and the supernatants were collected for the analyses of necrosis by Lactate dehydrogenase (LDH) assay (see below). The remaining cells were used to evaluate apoptosis by analyzing the formation of hypo-diploid nuclei. For this, the cells were detached and disrupted by scraping at 4°C in apoptosis' buffer containing 50 µg/ml EB, 0.01 g 0.01 g/L of sodium citrate, and 0.1% de Triton X-100. These samples were then analyzed by flow cytometry (Becton Dickinson, San Jose, CA) using a 488 nm argon laser and running under Cell Quest 3.3 software (BD biosciences). The events acquired for analysis (10.000/sample) were gated to eliminate cell aggregates and analyzed using WinMDI software (Version 2.8, Joseph Trotter, and The Scripps Researsh Institute La Jolla, USA).

Cell lyses (necrosis) was determined by measuring LDH enzymatic activity in the supernatants collected as above, using a commercially available colorimetric assay kit according to the maker's instructions (Doles, Goiania, GO, Brazil). Control lyses were obtained by using normal extracellular solution as 0% and non-infected cells treated with 0.1% Triton-X 100 as 100%.

### Dye uptake assays

ATPe-induced, dye uptake assays were performed as described [38]. Cells were first removed from the incubator, gently washed and kept at 37°C for 5 min, in normal extracellular solution. EB (10 µM final concentration), CF (5 mM), LY (3 mM), or SR-B (3 mM) and ATP (0–5 mM) were then added. These cells were kept under the same conditions for an additional period of 10 minutes and then gently washed in normal extracellular solution. Then cells were then incubated or not with Hoechst 33342 (1∶1000) for an additional 5 minutes. The dye uptake was determined by fluorescence microscopy using an Axiovert 100 microscope (Karl Zeiss, Oberkochen, Germany) equipped with an HBO lamp, an Olympus digital camera (Olympus American Inc., PA, USA) and Image-pro plus v 6.2 software (Media Cybernetics, Inc. Bethesda, MD, USA). Quantitative spectrofluorimetry was performed in experiments with CF and SR-B using an FLX-800 plate reader (BioTek Instruments Inc., Winooski, VT, USA) according to the following protocol [38]: The cells were gently washed five times with PBS, lysed by the addition of 100 µl PBS containing 0.01% BSA and 0.05% Triton X-100, scraped off the plate, and used for fluorescence determination using the following excitation and emission wavelengths ranges (nm): 420–450 and 528–520 for CF, and 516–520 and 620–640 for SR-B. Protein concentrations were determined by the Bradford method and a calibration curve was prepared for each dye in order to determine the final amount of dye taken up by the cells expressed as mg of dye/mg protein. The results were then normalized considering the uptake of uninfected macrophages treated with 5 mM ATP as 100%. In some experiments, freshly isolated intraperitoneal cells in suspension were exposed to ATP as above and dye uptake was measured by flow cytometry as previously described [39].

### Intracellular calcium measurements

The intracellular calcium concentration of macrophages was determined by Fura-2 fluorimetry as described [39]. Infected and non-infected macrophages prepared as described above in glass coverslips were loaded with 2.5 µM Fura-2-AM (Molecular Probes) for 40 min at room temperature in culture medium. The coverslips were then washed in PBS and mounted in a three-compartment perfusion chamber attached to the stage of an inverted microscope (NIKON DIAPHOT 300 TMD). Cells were perfused with PBS supplemented with 1 mM CaCl_2_ at 37°C at a rate of 1 ml/min. The intracellular calcium concentration of groups of a minimum of 20 cells was monitored continuously with the use of a fluorescence photometer (Photon Technology; Princeton, NJ). Fura-2 was excited alternately at 340 and 380 nm, and the emission at 510 nm was measured. The ratio measurement, which is proportional to the intracellular calcium concentration, was determined every 100 ms. ATP was perfused continuously while the temperature was kept constant at 37°C.

### Electron microscopy

In order to examine nucleotide treated cells using electron microscopy, uninfected and *L. amazonensis*-infected cells were incubated or not with ATP 3 mM for 30 min at 37°C in PBS, gently washed in PBS and incubated for an additional period of 8 hrs in complete DMEM medium at 37°C. The cells were then fixed in 2.5% glutaraldehyde, 4% paraformaldehyde in 0.1 M sodium cacodylate buffer containing 0.1 M sucrose and 3 mM CaCl_2_, pH 7.4 at room temperature for 60 min. After this, the cells were scraped off and transferred to an Eppendorf-tube for continued fixation overnight at 4°C. Following this procedure the cells were rinsed in PBS pH 7.4 and pelleted by centrifugation. These pellets were then postfixed in 1% osmium tetroxide dissolved in potassium ferrocianate for 1 hour; dehydrated sequentially in acetone; and embedded in Epon 812. Ultrathin sections (90 nm) were cut using LKB ultramicrotome and collected on copper grids. Contrast on the sections was obtained by uranyl acetate followed by lead citrate and examination performed in a Zeiss EM10C transmission electron microscope at 80 kV.

### Image acquisition and analysis

Differences between experimental groups were evaluated by the two-tailed unpaired Student's *t*-test. Each experiment was performed at least three times in duplicate. Data were analyzed using GraphPad InsTat software (GraphPad Software Inc., version 4.0). Values are mean ± s.d. The quantification of the fluorescence intensity was performed using the ImageJ 1.38X program (National Institute of Health, USA). Electron micrographs were digitalized employing an 8-bit gray scale at a resolution of 5.5 nm/pixel in an EPSON series Scanjet 3970.

## Results

### Cytotoxic effects of ATPe in macrophages and intracellular *L.amazonensis*

**Figure 1:**
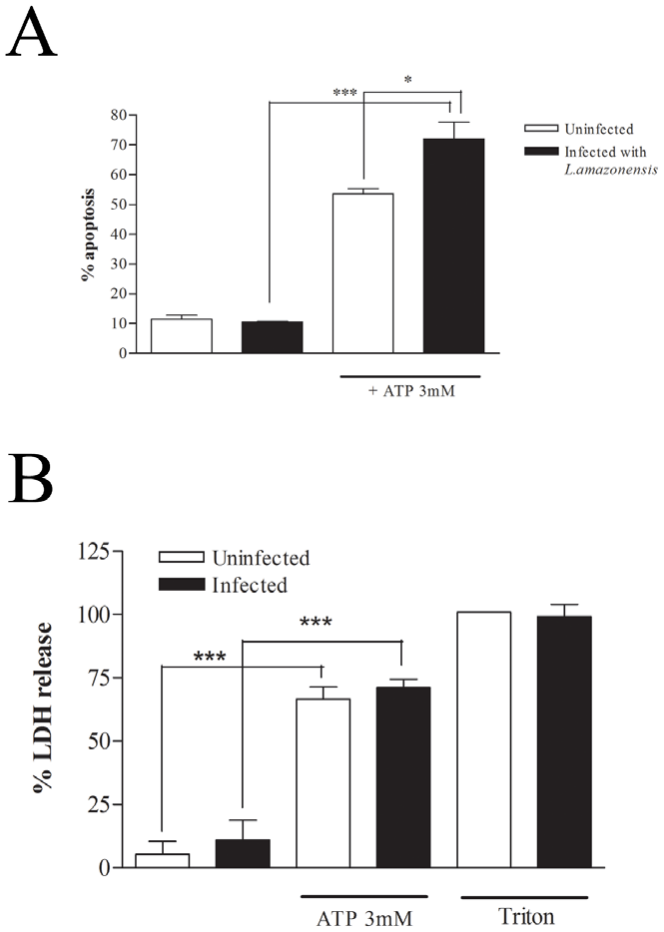
Extracellular ATP induces apoptosis and necrosis in *Leishmania*-infected macrophages. Resident macrophages were infected or not with *L.amazonensis* promastigotes at moi of 10∶1. After 4 hours free parasites were washed off and 24-hour post-infection cells were treated or not with 3 mM ATP for 30 min. (A) 10 hours after ATP treatment, cells were incubated with apoptosis buffer containing ethidium bromide and the nuclei were analyzed for DNA content by flow cytometry. (B) LDH activity measured in the supernatants of the same cells, collected before the addition of the apoptosis' buffer. n = 3. * P<0.05, ***P<0.001.

It is well established that ATPe, at millimolar concentrations, induces apoptosis and necrosis through the activation of P2X7 receptors in different cell types such as macrophages, dendritic cells and other cells [24], [40], [41]. We have recently shown that *L.amazonensis* infection up-regulates P2X7 expression and renders the cells more susceptible to ATPe-induced apoptosis and we have also shown that even at sub-lytic doses, ATPe can induce parasite elimination by macrophages [33]. At 3 mM, a condition that induces more than 50% of macrophages to undergo necrosis and apoptosis, a significant rise of 18±6% was observed in apoptosis (Fig. 1A) but not in necrosis (Fig. 1B) of infected cells. These results were confirmed by staining with annexin-V and propidium iodide 6 hrs after ATPe (data not shown).

**Figure 2:**
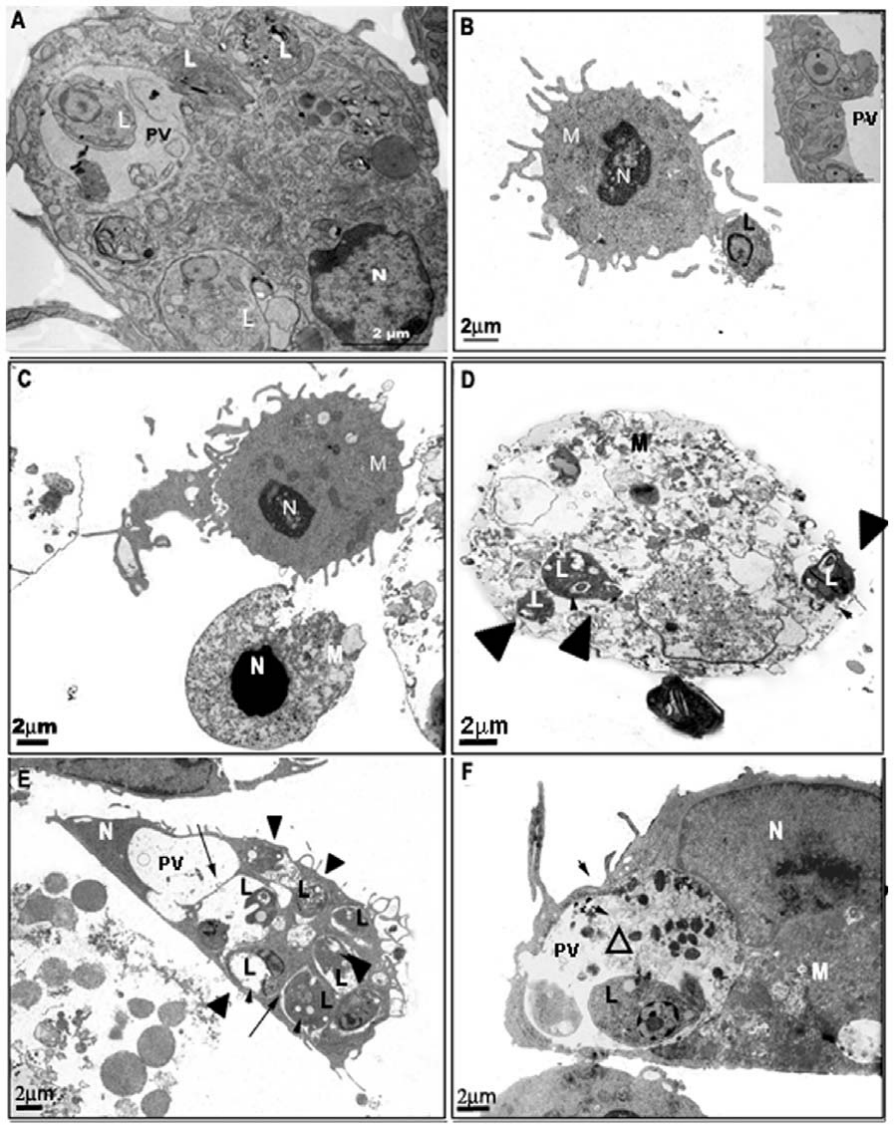
Ultrastructural analysis of ATPe-induced morphological damages in macrophages and intracellular *Leishmania*. Resident macrophages were infected with *L. amazonensis* promastigotes at moi of 10∶1. After 4 hours free parasites were washed off. 24-hour post-infection, cells were treated or not with 3 mM ATP for 30 min and 8 hours later fixed and processed for transmission electron microscopy. (A) Infected macrophage; (B) amastigote being internalized by an untreated macrophage, the insert shows amastigotes in the Parasitophorous vacuole; (C), (D), (E) and (F) Infected and ATPe-treated macrophages. M: macrophages, N: nucleus, L: *L. amazonensis*, Black arrows and *PV*: *L. amazonensis* Parasitophorous vacuole, Black arrowheads: Macrophages vacuole, Open triangle: degraded parasites, Black triangles: amastigotes presenting abnormal shapes with cytosolic vacuolation.

Cells were also observed by electron microscopy (Fig. 2). Infected macrophages with morphological alterations characteristic of apoptosis - condensed nuclei, decreased cytosolic area/volume, loss of cytosolic electron density, and difficult to identify organelles - were clearly observed after ATP treatment (Fig. 2C) as well as cells displaying typical characteristics of necrosis such as loss of shape, shrinkage, and loss of plasma membrane structure (Fig. 2D), characteristics not present in untreated cells (Figs. 2A–B). We also observed macrophages undergoing extensive vacuolation (Figs. 2 E–F). We observed that parasitophorous vacuoles (PV) of ATP-treated cells often appear to be fusionating to each other and with other vesicles (*arrows* in Fig. 2E). In some vacuoles we observed plasma membrane profiles and electrondense structures resembling degraded parasites (*open triangle* in Fig. 2F). In addition, we also observed loss of integrity of parasitophorous and parasite membranes, indicative of the induction of killing of both macrophages and parasites (*black triangles* in Fig. 2D). We also noticed the presence of amastigotes presenting abnormal shapes with cytosolic vacuolization (*black triangles* in Figs. 2E and 2D) if compared to amastigotes inside untreated cells (*L*) (Figs. 2A and 2B). These results are consistent with the strong induction of cell death of both macrophages and amastigotes induced by ATPe and the up-regulation of the expression of functional P2X7 receptors during the course of macrophage infection by *L.amazonensis*, as we have already described [33].

### Differential regulation of the uptake of anionic and cationic dyes by *L.amazonensis*

**Figure 3:**
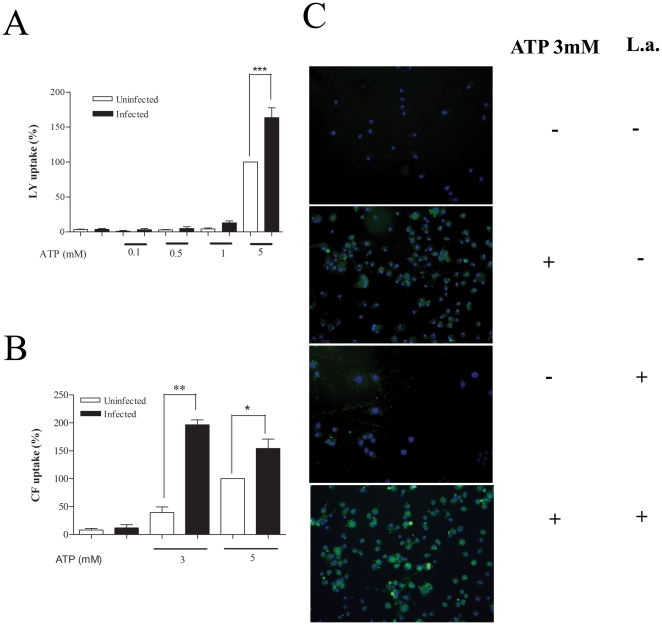
Increased uptake of anionic dyes by *L.amasonensis*-infected macrophages. Resident macrophages were infected or not with *L.amazonensis* promastigotes at moi of 10∶1. After 4 hours free parasites were washed off and 24-hour post-infection cells were treated or not with 3 mM ATP for 15 min. LY (A) or CF (B and C) was added 5 min after the addition of ATP. Cells were then washed 3 times with buffer and counted using fluorescence microscopy (C) or lysed for fluorimetric analysis (A and B) *n = 3*. Macrophage nuclei are stained in blue (Hoechst). Calibration Bar: 10 µm. * P<0.05, **P<0.01, ***P<0.001.

**Figure 4:**
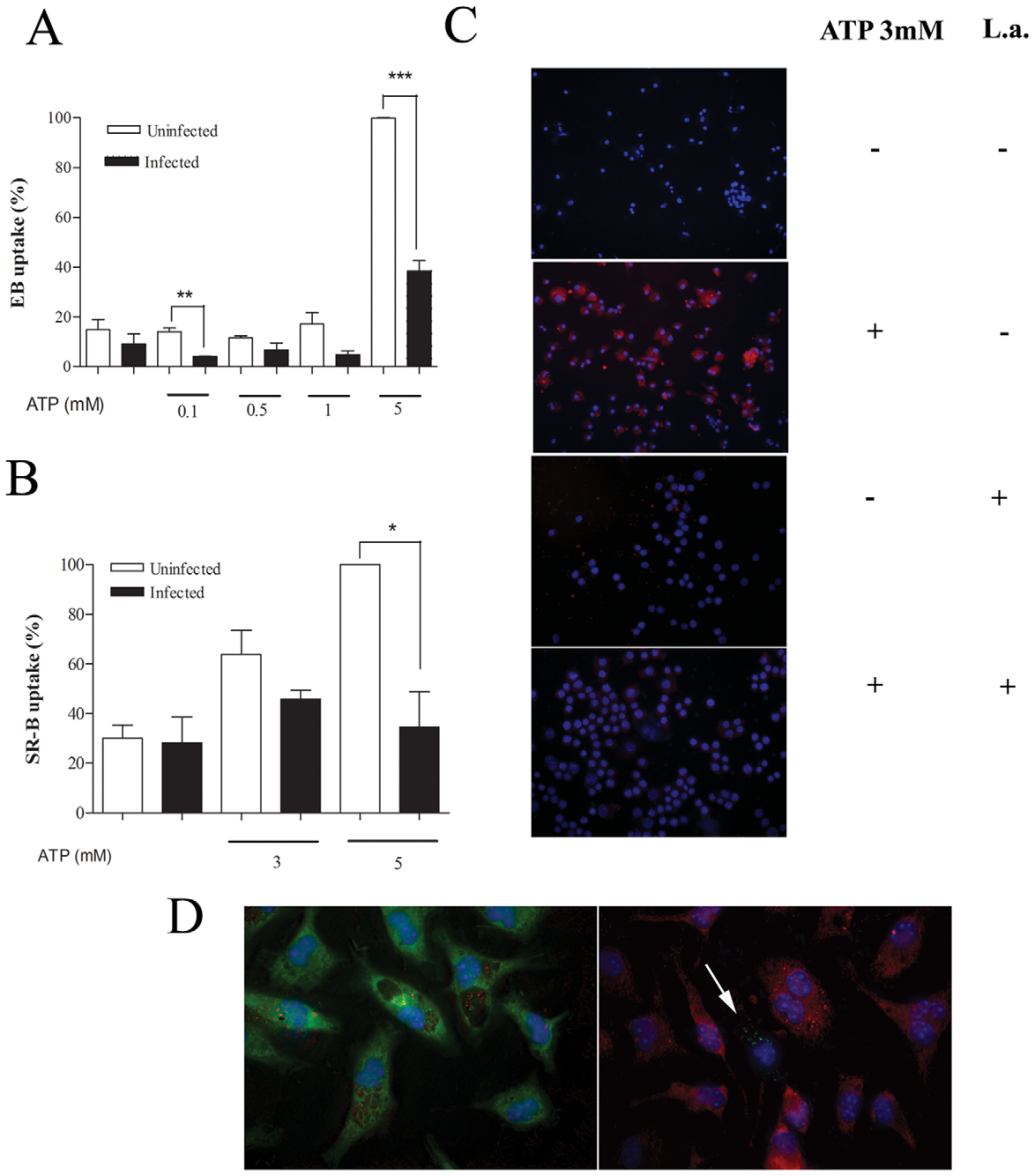
Decreased uptake of cationic dyes by *L.amasonensis*-infected macrophages. Resident macrophages were infected or not with *L.amazonensis* promastigotes at a moi of 10∶1. After 4 hours free parasites were washed off and 24-hour post-infection cells were treated or not with 3 mM ATP for 15 min. EB (A), SR-B (B, C, and right panel in D), and CF (left panel in D) was added 5 min after the addition of ATP. Cells were then washed 3 times with buffer and counted using fluorescence microscopy (C, D) or lysed for fluorimetric analysis (A and B) *n = 3*. Macrophage nuclei are stained in blue (Hoechst). White arrow in (D) indicate infected macrophage without SR-B uptake. Parasites are stained in red (D, left panel) or green (D, right panel) Calibration Bar: 10 µm. ** P<0.05, ***P<0.001.

The recent demonstration that ATPe can induce at least two distinct dye-uptake pathways for cationic and anionic fluorescent dyes respectively [37], [38] prompted us to investigate the possibility of differential modulation of these pathways in infected cells. In agreement with our own previously published data [33], macrophages infected with *L.amazonensis* displayed an enhanced ATPe-induced uptake of LY (Fig. 3A), the prototype anionic dye used to characterize P2X7-associated permeabilization, and also of CF (Figs. 3B–C), when compared with uninfected cells. The uptake of CF in both infected and non-infected macrophages was inhibited by pre-incubating the cells for 1 h with 300 uM oxATP (data not shown), a result that indicates that that in both conditions the ATPe-induced dye uptake is P2X7-dependent phenomena [40]. In marked contrast with these data, infected macrophages displayed a significant decrease in uptake of the cationic dyes EB, one of the dyes most frequently used to probe for functional P2X7 receptors (Fig. 4A) and SR-B a dye that contains both negative and positive charges (Figs. 4B–C). We also observed that the background in fluorescence is higher for cations (Fig. 4A and 4B) and that 12% of infected cells permeabilize to cationic dye spontaneously, a value not changed significantly by the presence of ATPe. (data not shown).

A more detailed observation of the infected macrophages after the uptake of SR-B indicated that only not infected cells could take this dye up. In order to better investigate this, promastigotes of *L.amazonensis* were stained with Dextran-FITC or Dextran-Rhodamine before infection and the ATP-induced dye-uptake assays were performed with SR-B and CF respectively (Fig. 4D). The results showed that CF uptake occurred in both infected and non-infected cells present in the same culture plat,(Fig. 4D, left panel), while the uptake of SR-B was abrogated only in infected cells, remaining normal in the non-infected cells (Fig. 4D, right panel). Interestingly, besides these differences, the typical morphological changes and bleb formation observed after ATPe treatment can be readily observed in both infected and uninfected macrophages (data not shown), consistent with the morphological changes observed using electron microscopy (Fig. 2).

In addition, we have extended our results to cells infected *in vivo*. Macrophages were collected 5 days after intraperitoneal injection of *L.amazonensis* and dye uptake assays were performed by flow cytometry. We observed that, similarly to macrophages infected *in vitro*, the uptake of CF was significantly increased and the uptake of SR-B was significantly inhibited in freshly isolated intraperitoneal cells derived from infected animals, (data not shown).

We next investigated the increase in free intracellular Ca^2+^ concentration upon the addition of ATPe to macrophages at 37°C, another hallmark of P2X7-associated signaling [23]. Macrophages display several P2X and P2Y receptors and, with the onset of continuous ATP stimulation, a biphasic signal that begins with a fast rise in the free intracellular calcium concentration, starting immediately upon the arrival of ATPe, followed by a sustained phase that can persist for several minutes, as long as ATPe concentration is maintained [23], [38], [39]. The second signal requires 37°C or near-physiological temperature and is solely mediated by P2X7 [23].

**Figure 5:**
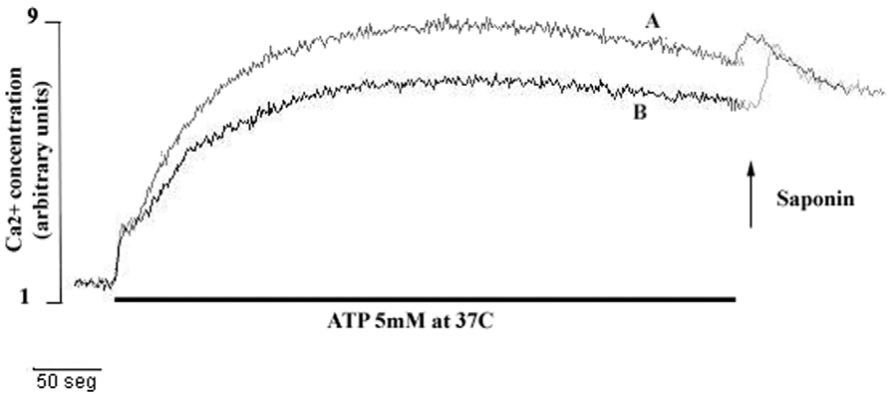
ATPe-induced Ca^2+^ signaling in *L. amazonensis*-infected macrophages. Resident macrophages were infected or not with *L.amazonensis* promastigotes at moi of 10∶1 in glass coverslips. After 4 hours free parasites were washed off. 48 hours later cells were loaded with Fura-2 and mounted on a perfusion chamber on the stage of a microscope for the measurement of the free intracellular Ca^2+^ concentration as described in Materials and Methods. Infected (A) and uninfected (B) macrophages cells were initially perfused with PBS supplemented with 1 mM CaCl_2_ and then continuously with 5 mM ATP for 500 s (black horizontal bar). Saponin was then added in a *bolus* to obtain 100% calcium load. Cells were maintained at 37°C throughout the experiment. Representative data of a single experiment are shown. Experiments were repeated at least three times with similar results.

We showed that both infected and non-infected macrophages responded to perfusion of 5 mM ATP at 37°C, with the typical P2X7-associated pattern consisting of a strong biphasic response (Fig. 5). In addition to having the same time pattern, the response of the infected cells consistently reached a slightly higher value.

### Dye uptake phenomenon is dependent on active manipulation of the host cell by *Leishmania*

**Figure 6:**
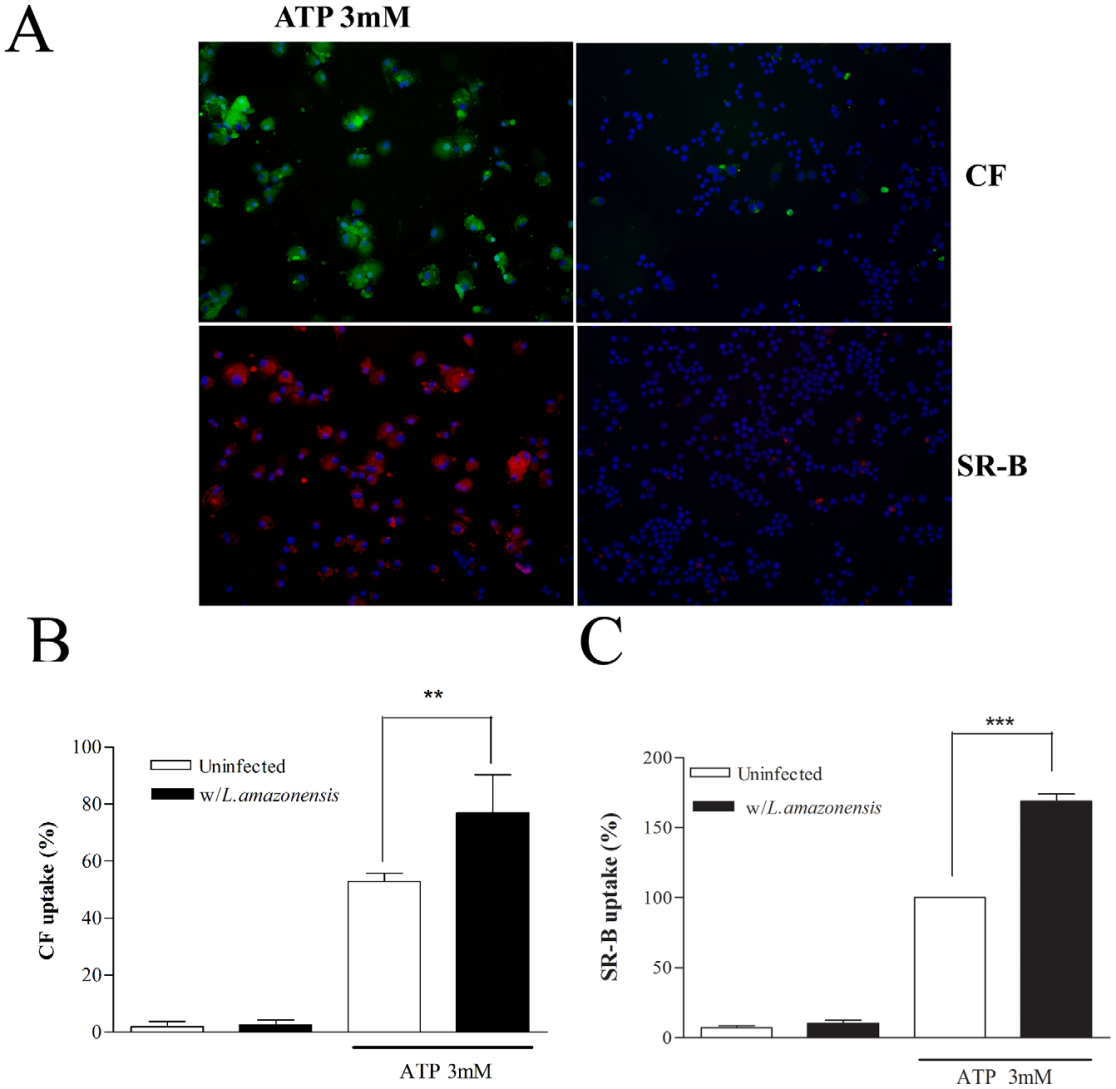
Modulation of the uptake of cationic dyes is dependent on parasite metabolism. Resident macrophages were exposed or not with dead *L.amazonensis* promastigotes at a concentration equivalent to a moi of 10∶1. After 4 hours free parasites were washed off and 24-hour post-interaction cells were treated or not with 3 mM ATP for 15 min. CF (A and B) or SR-B (A and C) was added 5 min after the addition of ATP. Cells were then washed 3 times with buffer and analyzed with fluorescence microscopy (A) or lysed for fluorimetric analysis (B and C) *n = 3*. Macrophage nuclei are stained in blue (Hoechst). Calibration Bar: 10 µm. ** P<0.01, ***P<0.001.

Since phagocytosis is the main pathway used by *Leishmania* to enter the macrophages (Reviewed by [42]) we questioned whether phagocytosis could be responsible for the differential modulation of the uptake of cationic and anionic dyes by itself. We then repeated the dye uptake experiments described above using parasites killed by freezing-and-thawing. We observed that under these experimental conditions macrophages displayed a significantly increased uptake of both anionic (Figs. 6A and 6B) and cationic dyes (Figs. 6A and 6B). We repeated these experiments using parasites fixed with paraformaldehyde and obtained similar results (data not shown). We also observed that, contrary to what happened with live parasites dead parasites are unable to up-regulate ATPe-induced apoptosis (data not shown).

## Discussion

Intracellular parasites use several mechanisms to exploit and subvert hosts function and escape the immune system and host cell defenses [10], [43]–[47] but even when the parasite has a powerful weapon to inhibit or to stimulate host function, the host may have alternative pathways that can be activated to eliminate the invading parasite. We and others have demonstrated that activation of P2X7 receptors of macrophages can induce the killing of intracellular microorganisms such as *Mycobacterium tuberculosis*, *Chlamydia*, *Toxoplasma* and *Leishmania* [25], [30], [33], [48]–[51]. It is therefore intriguing that some of these parasites, as is the case of *L.amazonensis*, are skilled disruptors of host cell apoptosis pathways [52]–[54] but can at the same time up-regulate the potentially pro-apoptotic P2X7 ATP receptors of macrophages [33]. During *L.amazonensis* infection, macrophages are exploited by producing nutrients that are acquired by the protozoan, inhibiting its microbicidal mechanisms like reactive oxygen and nitrogen species and maintaining a communal parasitophorous vacuole with phagolysosomal characteristics [55]. Once inside the host cells, *Leishmania* amastigotes actively regulate the entry of several molecules [56] and modulate signaling pathways and calcium homeostasis in the host cell [44], [57]. These data unveil a sophisticated control of metabolic and transport pathway that deserves further clarification. In order to further understand *Leishmania*-macrophage interaction, we re-addressed the studies of the phenomenon usually referred to as “ATPe-induced P2X7-mediated membrane permeabilization” taking into account recent findings that demonstrated that this phenomenon, usually studied by fluorescent dye uptake assays, is mediated by more than one transport mechanism [37], [38].

We started by confirming previous work showing that macrophages infected with *L.amazonensis* are more sensitive to ATPe, undergoing apoptosis at higher levels than uninfected cells, and that the parasites inside the treated macrophages are also damaged, indicating that they can undergo death even before the macrophage itself. We also observed morphological changes typical of vacuolization in infected macrophages treated with ATPe, as previously described by others [58]. In addition, ATPe also induced a sustained increase in free intracellular Ca^2+^ concentration in both infected and uninfected macrophages maintained in the presence of ATPe for several minutes at 37°C, one of the hallmarks of the “permeabilization” phenomenon [23].

ATPe-induced macrophage death, parasite killing, vacuolization, and Ca^2+^ signaling have all been associated with the expression of P2X7 receptors [23], [33], [58], known to be up-regulated during macrophage infection by *L.amazonensis* [33]. Consistent with these observations we have also shown that uptake of LY and CF are up-regulated in infected macrophages, a phenomenon that has been associated with the formation of a permeation pore [23], [38], [59], [60], possibly due to pannexin molecules in the membrane [35], [36]. Surprisingly, we show here that the uptake of the cationic dyes EB and SR-B is down-regulated during infection. This result not only confirms that cationic dyes can be taken up by a different P2X7-activated transport mechanism in macrophages [37], [38] but also demonstrates for the first time that these transport mechanisms can be differentially modulated in immunologically relevant conditions.

It is therefore reasonable to state that only the transport mechanism accessed by the anionic dyes the LY and CF is involved in macrophage death, vacuolization, blebbing, Ca^2+^ signaling, and parasite killing. It is also clear that, at least in infected macrophages, the mechanism of transport involved in the ATP-induced uptake of cationic dyes is not important for triggering neither of these phenomena. However, the precise role of the anionic and the cationic branches of the ATP-induced transport mechanisms in each of the many P2X7-dependent cellular responses needs to be better characterized. This is the case of apoptosis. We showed that infected cells display increased ATP-induced apoptosis and increased uptake of anionic dyes (Figs. 1 and 3). However, macrophages exposed dead parasites also up-regulates the uptake of anionic dyes (Fig. 6), without the corresponding increase in apoptosis (not shown). One possibility to explain this apparent contradiction is may be the involvement of an active participation of the live parasites. Further experiments are needed to investigate this possibility.

The mechanism involved in EB and SR-B uptake remains elusive as well as the pathway used by the parasites to down-regulate it. At the present moment we can only say that this down-regulation requires the active participation of live parasites. Further experiments are required to clarify the nature of this transport mechanism, its role in *Leishmania* infection and the mechanism used by the parasite to induce its down-regulation during infection.

The study of the selectivity of each of these transport mechanisms it beyond the scope of this study, but our results give us some interesting hints. We have previously shown that the Z-pores possibly involved in the permeation of LY and CF is also non-selective for small cations and anions, including Ca^2+^, Tris, and Glutamate [38], [60] but it is not clear how larger organic cations could be sorted out from this permeation pathway and enters the cells though an as yet unidentified, non-difusional pathway. Interestingly, since the results of ATP-induced uptake of SR-B, a sulfonated molecule that displays both positive and negative charges, are similar to the ones obtained with EB and other dyes that display only positive charges [38], it is possible to assume that the presence of a positively charged group is the dominant condition required to direct the dye to this pathway. In this regard, it is interesting to notice that the infection of macrophages with *L.amazonensis* is an interesting tool to help uncover the molecular nature of this cationic transport mechanism.

The results presented here suggest that the decreased uptake of cationic dyes is due to the down-modulation of this transport pathway by *L.amazonensis* infection. However, other possibilities, such as an increase in the efflux of cationic dyes may also explain these results and more experiments will be required to investigate the mechanism of decrease in the uptake of cationic dyes.

Regarding the P2X7 receptors and the transport mechanisms associated with the uptake of Ca^2+^, LY and CF, it seems contradictory that the parasites can induce the up-regulation of a mechanism that could ultimately lead to its elimination. We should however mention that apoptosis has been proposed to be part of the survival/escape strategy of *Leishmania* [45], [61]. The phagocytosis of apoptotic bodies of infected macrophages could not only induce immune tolerance but also help propagate the infection. In addition, the activation of P2X7-associated phenomena must be under some type of control by the parasite in order to avoid over activation or activation at an unfavorable moment of the *Leishmania* life cycle. In this regard it is interesting to note that recent findings indicate that *L.amazonensis* expresses ecto-nucleotidase activity and its level of activity correlates with macrophage infectivity and size of lesion [62], [63], suggesting that degradation of ATPe may be used by the parasites to avoid early activation of the P2X7-associated pathways.

Another indication that the parasite induces some kind of down-regulation of the ATPe-induced permeabilization pore even in the presence of higher levels of P2X7 receptors comes from electrophysiological studies of K^+^ channels in infected macrophages. It has recently been demonstrated that after 24 h infection with *L.amazonensis* macrophages display an increased activation of K^+^ channels and a more polarized membrane potential [64], a condition that favors the closed state of the Z pores [38], [60].

We therefore conclude that a successful infection of macrophages by the parasite requires a fine balance between the up or down-regulation of some P2X7-associated phenomena and their respective regulatory mechanisms. This balance seem to be important also for *in vivo* infection since the same modulation of dye uptake was observed when macrophages were infected *in vivo* (not show).

The significance of the down-regulation of the mechanism of uptake of cationic dyes is still to be elucidated. Interestingly, under our experimental conditions we also observed that 12% of macrophages of the infected cultures displayed spontaneous uptake of cationic dyes regardless of the presence of ATPe. A more detailed observation of this phenomenon using fluorescently stained *L.amazonensis* (Fig. 4D), lead us to the observation that the down-modulation of the uptake of cationic dyes in each cell requires the presence of an active infection, suggesting that the blockade of this branch of the P2X7-induced pathway is necessary for the parasite survival. A better characterization of this pathway and its potential role in parasite killing deserves better clarification in the future.

In addition to the significance of these observations regarding host-parasite interactions, our results can also help us to better understand the differences between the cationic and anionic transport mechanism and their relationships with other ATPe-associated phenomena. In this regard, our results confirm that macrophages do display more than one mechanism involved in the so-called “ATPe-induced P2X7-mediated membrane permeabilization phenomenon”. The possibility that differential regulation of the uptake of cationic and anionic dyes may occur implies that we should be cautious with the interpretation of much published data using only one signal (e.g. the uptake of LY, uptake of EB or YoPro-1 or calcium signaling) to access the “permeabilization phenomenon” and the functional expression of P2X7 receptors, as is most frequently the case in the available literature. This may be particularly relevant when planning new experiments using macrophages and other cells of the immune system, opening new possibilities in nucleotide-based immune interventions.
